# The Impact of Hypoxic Hepatitis on Clinical Outcomes after Extracorporeal Cardiopulmonary Resuscitation

**DOI:** 10.3390/jcm9092994

**Published:** 2020-09-16

**Authors:** Yun Im Lee, Min Goo Kang, Ryoung-Eun Ko, Taek Kyu Park, Chi Ryang Chung, Yang Hyun Cho, Kyeongman Jeon, Gee Young Suh, Jeong Hoon Yang

**Affiliations:** 1 Department of Critical Care Medicine, Samsung Medical Center, Sungkyunkwan University School of Medicine, Seoul 06351, Korea; yunim.lee@samsung.com (Y.I.L.); mingook.kang@samsung.com (M.G.K.); ryoungeun.ko@samsung.com (R.-E.K.); icu.chung@samsung.com (C.R.C.); kyeongman.jeon@samsung.com (K.J.); gy.suh@samsung.com (G.Y.S.); 2 Division of Cardiology, Department of Medicine, Samsung Medical Center, Sungkyunkwan University School of Medicine, Seoul 06351, Korea; taekkyu.park@samsung.com; 3 Department of Thoracic and Cardiovascular Surgery, Samsung Medical Center, Sungkyunkwan University School of Medicine, Seoul 06351, Korea; yanghyun.cho@samsung.com; 4 Division of Pulmonary and Critical Care Medicine, Department of Medicine, Samsung Medical Center, Sungkyunkwan University School of Medicine, Seoul 06351, Korea

**Keywords:** extracorporeal cardiopulmonary resuscitation, hypoxic hepatitis, hypoxic liver injury

## Abstract

Although there have been several reports regarding the association between hypoxic hepatic injury and clinical outcomes in patients who underwent conventional cardiopulmonary resuscitation (CPR), limited data are available in the setting of extracorporeal CPR (ECPR). Patients who received ECPR due to either in- or out-of-hospital cardiac arrest from May 2004 through December 2018 were eligible. Hypoxic hepatitis (HH) was defined as an increased aspartate aminotransferase or alanine aminotransferase level to more than 20 times the upper normal range. The primary outcome was in-hospital mortality. In addition, we assessed poor neurological outcome defined as a Cerebral Performance Categories score of 3 to 5 at discharge and the predictors of HH occurrence. Among 365 ECPR patients, 90 (24.7%) were identified as having HH. The in-hospital mortality and poor neurologic outcomes in the HH group were significantly higher than those of the non-HH group (72.2% vs. 54.9%, *p* = 0.004 and 77.8% vs. 63.6%, *p* = 0.013, respectively). As indicators of hepatic dysfunction, patients with hypoalbuminemia (albumin < 3 g/dL) or coagulopathy (international normalized ratio > 1.5) had significantly higher mortalities than those of their counterparts (*p* = 0.005 and *p* < 0.001, respectively). In multivariable logistic regression, age and acute kidney injury requiring continuous renal replacement therapy were predictors for development of HH (*p* = 0.046 and *p* < 0.001 respectively). Furthermore age, arrest due to ischemic heart disease, initial shockable rhythm, out-of-hospital cardiac arrest, lowflow time, continuous renal replacement therapy, and HH were significant predictors for in-hospital mortality. HH was a frequent complication and associated with poor clinical outcomes in ECPR patients.

## 1. Introduction

Utilization of extracorporeal membrane oxygenation (ECMO) in cardiopulmonary resuscitation (CPR), called extracorporeal cardiopulmonary resuscitation or ECPR, is currently increasing [1,2]. It is well-known that organ failures after CPR affect the prognosis of post cardiac arrest survivors. In particular, previous studies showed that hepatic injury was associated with poor clinical outcomes in patients who received conventional CPR [3,4,5]. To evaluate this relationship, several biomarkers such as bilirubin, albumin, prothrombin time, and aminotransferases were used as indicators of hepatic dysfunction [6]. Among them, aminotransferases are sensitive to hepatocellular injury and increase shortly after hypoxic damage, while serum bilirubin or albumin level needs more time to change. Since aminotransferases may be more appropriate biomarkers to reflect hypoxic damage in patients with cardiac arrest, hypoxic hepatitis (HH) has been defined as a rapid increase of aminotransferase levels in an acute setting of cardiac, circulatory, or respiratory failure [7]. However, in the setting of ECPR, limited data are available about the association between hypoxic liver injury and clinical outcomes. Therefore, we aimed to investigate the incidence, risk factors, and prognosis of HH in patients who underwent ECPR.

## 2. Methods

### 2.1. Study Population

**Figure 1:**
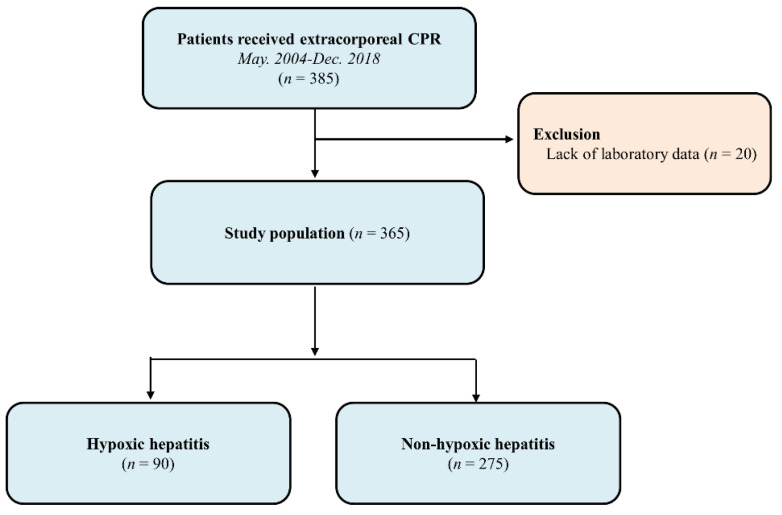
Study flow chart.

This is a retrospective, single-center, observational study. Adult patients who were admitted to the intensive care units in our tertiary hospital (Samsung Medical Center, Seoul, and Republic of Korea) after ECPR due to either in- or out-of-hospital cardiac arrest from May 2004 to December 2018 were eligible. Patients who were 18 years old or older at the time of arrest were included in the study. Patients with inefficient data to evaluate liver function were excluded. For the purpose of the current analysis, enrolled patients were stratified into HH and non-HH groups (Figure 1). This study was approved by the Institutional Review Board of Samsung Medical Center (2019-10-119). The requirement for informed consent was waived due to the retrospective nature of the study.

### 2.2. Data Collection, Definitions, and Outcomes

Baseline characteristics of comorbidities, management in intensive care units, and details on ECPR were collected retrospectively through medical record review. When the same biologic markers were measured several times before ECMO insertion, the laboratory value measured at the time nearest ECMO insertion was recorded.

HH was defined as an increased aspartate aminotransferase (AST) or alanine aminotransferase (ALT) level to more than 20 times the upper normal range (AST ≤ 40 IU/L and ALT ≤ 41 IU/L in our hospital) from day 0 to day 2, i.e., AST > 800 IU/L or ALT > 820 IU/L [4,8]. Chronic liver disease was defined as radiologic or serologic evidence of liver cirrhosis or chronic hepatitis. Radiologic modalities included computed tomography, magnetic resonance imaging, and ultrasound. Positive hepatitis B surface antigen and anti-hepatitis C virus antibody were regarded as serologic evidence of chronic viral hepatitis. ECPR was defined by successful veno-arterial ECMO implantation and pump-on with external chest compression during the index procedure in patients with cardiac arrest. When return of spontaneous circulation occurs during ECMO cannulation, practitioners typically do not remove the cannula or stop the ECMO pump-on process. CPR to ECMO pump-on time was defined as the time from initiation of chest compressions to the time of ECMO pump activation [9,10].

The cut-off values of the markers for hepatic dysfunction were set based on previous publications. For instance, the reference point of total bilirubin is based on simplified acute physiology score III and Child–Pugh score [11,12,13]. In addition, bilirubin of 2 mg or above meets the criterion of organ dysfunction in “Sepsis-3” guidelines, which is acute change in sequential organ failure assessment score ≥ 2 points [14]. The reference point of 1.5 in international normalized ratio (INR) was based on the definition of acute liver failure [15].

The primary outcome was in-hospital mortality. In addition, we assessed poor neurological outcome, which was defined as Glasgow-Pittsburgh cerebral performance categories score of 3 to 5 at discharge and the predictors of HH occurrence.

### 2.3. Statistical Analysis

Categorical variables were presented as number and relative frequency, and their group differences were compared using the Chi-square test or Fisher’s exact test, as appropriate. Continuous variables were presented as mean ± standard deviation or median (25th to 75th percentiles), and differences were compared using Student’s *t*-test or Wilcoxon rank-sum test when applicable. Cumulative incidences of mortality were calculated by Kaplan-Meier estimates and compared using a log-rank test. To estimate odd ratios for in-hospital mortality, we used a logistic regression model. Variables included in the univariable analysis were age, sex, body weight, comorbidities, smoking, and utilization of organ support modalities such as mechanical ventilator, continuous renal replacement therapy (CRRT), and vasopressors. Variables with *p* < 0.2 in univariable analysis and those thought to be clinically relevant were included in multivariable logistic regression. We also computed odds ratios with 95 % confidence interval (CI). Statistical analyses were performed with IBM SPSS Statistics software Version 25.0 for Windows (IBM, Armonk, NY, USA), GraphPad Prism 8 (GraphPad Software, San Diego, CA, USA), and MedCalc 19.2.0. (MedCalc Software, Ostend, Belgium). All tests were two-tailed, and *p* < 0.05 was considered statistically significant.

## 3. Results

### 3.1. Baseline Characteristics

Three hundred sixty-five patients were finally analyzed in this study. Of them, 90 (24.7%) were identified as belonging to the HH group and 275 (75.3%) as belonging to the non-HH group. Baseline characteristics are presented in Table 1. The median age of the patients was 61 (51–71) years. Two hundred fifty-two (69.0%) patients were male, and 26 patients (7.1%) had chronic liver disease. The incidence of chronic liver disease was not significantly different between the two groups. Most of the patients suffered in-hospital cardiac arrest (86.8%). Those in the non-HH group had higher incidences of chronic kidney disease and out-of-hospital cardiac arrest compared with those of the HH group. The HH group had a longer duration of ECMO application and a higher incidence of CRRT than non-HH group. The CPR to ECMO pump-on time was not significantly different between the two groups [HH group: 35 (20–53) minutes and non-HH group: 30 (20–47) minutes, *p* = 0.373]. The results of the laboratory tests according to the time of ECPR in HH group are presented in Table S1.

### 3.2. Hepatic Dysfunction and Mortality

**Figure 2:**
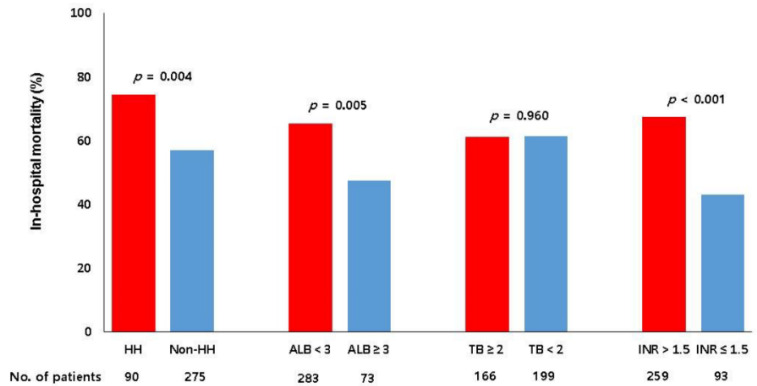
Relationship between hepatic dysfunction and in-hospital mortality. HH, hypoxic hepatitis; ALB, albumin (g/dL); TB, total bilirubin (mg/dL); INR, international normalized ratio.

The relationship between hepatic dysfunction and in-hospital mortality according to various biomarkers is shown in Figure 2. The mortalities of patients with albumin level below 3 g/dL or INR above 1.5 were significantly higher than those of their counterparts (*p* = 0.005 and *p* < 0.001 respectively). The mortality of patients with total bilirubin 2 mg/dL or above was not significantly different compared with that of their counterparts (*p* = 0.960).

### 3.3. Clinical Outcomes

**Figure 3:**
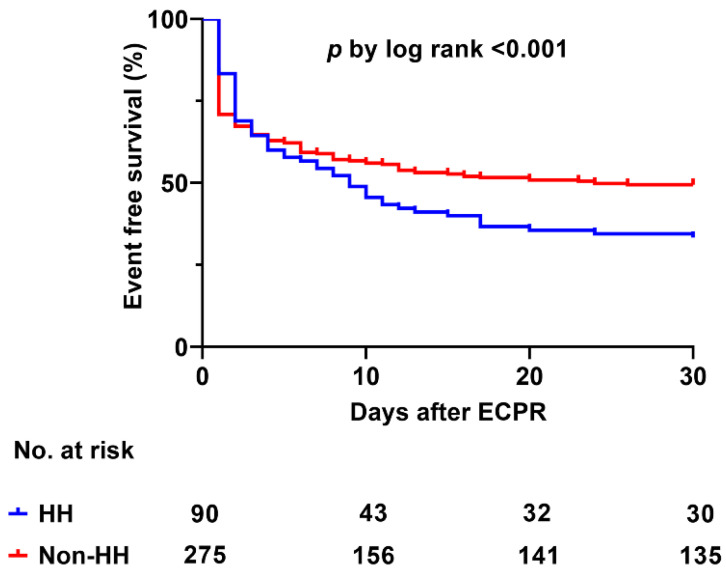
Kaplan-Meier curve for 30-day mortality between HH and non-HH groups. HH, hypoxic hepatitis.

Sixty-five patients (72.2%) from the HH group and 151 patients (54.9%) from the non-HH group died in the hospital (*p* = 0.004). Moreover, 70 patients (77.8%) from the HH group and 175 patients (63.6%) from the non-HH group showed poor neurological outcomes (*p* = 0.013). Eleven patients needed left ventricle unloading strategies in the study population (4 in the HH group and 7 in non-HH group). Among them, nine patient underwent percutaneous trans-septal left atrial cannulation and two patients underwent surgical decompression. The composite incidences of complications associated with ECMO were significantly different between two groups (Table S2). The Kaplan-Meier curves demonstrated that the HH group had a higher incidence of 30-day mortality compared with the non-HH group (*p* < 0.001) (Figure 3). Among the 26 patients with chronic liver disease, in-hospital mortality was not significantly different between the two groups (HH group: 66.7% and non-HH group: 70%, *p* = 0.999). In multivariable logistic regression, age and acute kidney injury requiring CRRT were predictors for development of HH (*p* = 0.046 and < 0.001 respectively) (Table 2). Furthermore, age, arrest due to ischemic heart disease, initial shockable rhythm, out-of-hospital cardiac arrest, CPR to ECMO pump-on time, CRRT, and HH were significant predictors for in-hospital mortality (Table 3).

## 4. Discussion

The current study aimed to evaluate the associations between hepatic dysfunction and clinical outcomes in a relatively large number of ECPR cases. The major findings of this study were as follows: (1) HH was a frequent complication after ECPR; (2) various biomarkers for hepatic dysfunction such as low albumin and prolonged INR were associated with in-hospital mortality, but bilirubin was not; (3) age and acute kidney injury requiring CRRT were predictors for development of HH after ECPR; and (4) HH was associated with increased in-hospital mortality and poor neurological outcomes.

Until now, no controlled trials have been performed to determine the optimal approach to evaluate abnormal liver tests even for the general population, although there are numerous studies and guidelines on abnormal liver chemistries [16]. Accordingly, hepatic dysfunction cannot be determined by only one laboratory value but in context with other biological markers and clinical information. Nevertheless, some biomarkers are generally thought to be more closely associated with specific hepatic function than others are. Serum bilirubin is considered as representing hepatic conjugation and excretion function. However, serum bilirubin peaks later than aminotransferases and can increase in cholestasis, which is common in critically ill patients [17,18]. Serum albumin has a limited role in the acute setting because its circulating half-life is three weeks. Moreover, prothrombin time is affected by anticoagulation therapy, which is inevitable in veno-arterial ECMO. On the other hand, aminotransferases increase shortly after hypoxic damage and decrease rapidly due to their short half-lives [7,19,20]. This feature makes aminotransferases more appropriate to evaluate hepatic dysfunction in patients with cardiac arrest. Accordingly, in previous studies, HH was defined as rapid increase of aminotransferase levels and was associated with mortality in critically ill patients [21,22]. In this study, AST and lactate dehydrogenase were higher than ALT in substantial patients with HH and these findings can help distinguish between ischemic and other causes of liver injury. These biochemical features are consistent with those of previous studies [21,23,24].

Previous studies reported that the prevalence of HH in conventional CPR ranged from 7% to 21% [3,4,5,8]. In a recent study that analyzed the outcomes of post cardiac arrest survivors, the researchers delineated that hypoxic liver injury was associated with unfavorable neurological outcome and one-year mortality after adjusting for multiple variables such as age, low flow time, and sequential organ failure assessment score [3]. Champigneulle et al. reported the association between HH and increased intensive care unit mortality in patients with out-of-hospital cardiac arrest [4]. However, in the setting of ECPR, there was no study regarding the association between HH and clinical outcomes. Thus, we investigated the prognostic implication of HH in patients who underwent ECPR and found that HH was associated with in-hospital mortality and poor neurological outcomes.

In previous studies, the factors associated with occurrence of HH were male sex, shockable rhythm, Charlson comorbidity index, cardiac failure, and time from collapse to return of spontaneous circulation [3,4]. In this study, age and acute kidney injury requiring CRRT were significant factors associated with HH, and age had a negative association with occurrence of HH. We assume that some older patients might have had HH even though their aminotransferase level was less than 800 IU/L, which was the cutoff value of HH in this study because the upper normal limit of aminotransferase declines after middle age [20,25,26]. This may have influenced the impact of age on HH occurrence. Renal impairment after cardiac arrest is quite common, and acute kidney injury requiring CRRT may represent the severity of hypoxic damage through the same mechanism of occurrence of HH. In previous studies, low flow time was a significant predictor of HH in conventional CPR [3,4,5,8], while low flow time measured as CPR to ECMO pump-on time was not a significant predictor of HH although it was a significant prognostic factor for in-hospital mortality in this study. This discrepancy might be caused by differences between CPR with and without ECMO. Full cardiac support is possible after pump-on of ECMO, while organ hypoperfusion remains even after return of spontaneous circulation in some patients that received conventional CPR. In addition, the proportion of out-of-hospital cardiac arrest in the study population might influence the impact of low flow time on occurrence of HH. The rate of out-of-hospital cardiac arrest included in previous studies ranged from 55% to 100% [3,4,5,27]. However, it was only 13.2% in this study. For out-of-hospital cardiac arrest, actual low flow time could be longer than recorded. Moreover, if a bystander initiated CPR, cardiac compression might be less effective than that provided by health professionals. In the same context, the only clinically modifiable factors of survival to discharge and the occurrence of HH are the qualities of CPR and CPR to pump-on time while age, initial arrest rhythm, and arrest location are not modifiable factors. Thus, we suggest that a multidisciplinary CPR team including a well-organized rapid response on-site ECMO team is needed to improve the outcomes in the setting of ECPR.

There are a few limitations in this study. First, there could be veiled liver diseases in some patients that could have affected the level of aminotransferases because not every patient received radiologic examination for underlying liver disease. In addition, we did not assessed the noninvasive markers associated with hepatic fibrosis such as aspartate aminotransferase-to-platelet ratio index. Second, drug-related hepatic toxicity or muscular injury could have affected the levels of aminotransferases. In the setting of ECPR, elevation of muscle enzymes can occur for several reasons such as ischemic injury, external chest compression and limb ischemia. Elevated AST along with elevated muscle enzyme could be confused with HH, particularly in patients undergone long-lasting external chest compression, which could cause extensive muscle injury. Third, the CPC score was determined based on medical records because of the design of this study. Fourth, this study was conducted using data gathered over a long period of time. During that period, there were many changes in post-arrest management that may have affected the outcomes. Fifth, the laboratory tests were not taken on the same time interval from the time of ECPR. Finally, selection bias influencing the results is possible because this is a retrospective, observational study conducted in a single center.

## 5. Conclusions

HH was a frequent complication after ECPR and was associated with increased in-hospital mortality and poor neurological outcomes at discharge.

## Figures and Tables

**Table 1:** Baseline characteristics.

	HH (*n* = 90)	Non-HH (*n* = 275)	*p* Value
Patient demographics			
Age (year)	59.5 (47–71)	62 (52–72)	0.203
Gender, male	67 (74.4%)	185 (67.3%)	0.201
Weight (kg)	66 (57.2–75.8)	65 (57.3–72.2)	0.360
Smoking	31 (34.8%)	95 (34.8%)	0.995
Comorbidities			
Diabetes mellitus	28 (31.1%)	94 (34.2%)	0.592
Hypertension	36 (40.0%)	141 (51.3%)	0.063
Malignancy	18 (20.0%)	40 (14.5%)	0.219
Dyslipidemia	11 (12.2%)	35 (12.7%)	0.900
Chronic kidney disease ^a^	6 (6.7%)	41 (14.9%)	0.043
Chronic liver disease	6 (6.7%)	20 (7.3%)	0.846
Previous coronary artery disease	16 (17.8%)	51 (18.5%)	0.870
Arrest due to ischemic heart disease	38 (42.2%)	142 (51.6%)	0.121
Initial shockable rhythm	22 (24.4%)	95 (34.5%)	0.075
Out-of-hospital cardiac arrest	6 (6.7%)	42 (15.3%)	0.036
ECPR details			
CPR to ECMO pump-on time (min)	35 (20–53)	30 (20–47)	0.373
ECMO duration (day)	3 (1–6)	1 (0–3)	<0.001
In-hospital management			
Mechanical ventilator	82 (91.1%)	232 (84.4%)	0.109
CRRT	54 (60.0%)	83 (30.2%)	<0.001
Vasopressor	88 (97.8%)	255 (92.7%)	0.081

^a^ Chronic kidney disease is defined as either kidney damage or GFR <60 mL/min/1.73 m^2^ for ≥3 months; CPR, cardiopulmonary resuscitation; ECPR, extracorporeal cardiopulmonary resuscitation; ECMO, extracorporeal membrane oxygenation; CRRT, continuous renal replacement therapy.

**Table 2:** Factors associated with occurrence of hypoxic hepatitis.

	* Adjusted OR (95% CI)	*p* Value
Age (year)	0.983 (0.966–0.999)	0.046
Gender, male	1.503 (0.839–2.691)	0.171
Chronic liver disease	0.868 (0.310–2.429)	0.788
Arrest due to ischemic heart disease	0.955 (0.544–1.675)	0.873
Initial shockable rhythm	0.605 (0.333–1.098)	0.098
Out-of-hospital cardiac arrest	2.449 (0.950–6.313)	0.064
CPR to ECMO pump-on time ≥ 30 min	1.608 (0.944–2.736)	0.080
CRRT	3.518 (2.103–5.885)	<0.001

CPR, cardiopulmonary resuscitation; ECMO, extracorporeal membrane oxygenator; CRRT, continuous renal replacement therapy; * adjusted with hypertension, chronic kidney disease, mechanical ventilator and vasopressor.

**Table 3:** Factors Associated with In-hospital Mortality after ECPR.

	* Adjusted OR (95% CI)	*p* Value
Age (year)	1.033 (1.015–1.051)	<0.001
Arrest due to ischemic heart disease	0.517 (0.301–0.889)	0.017
Initial shockable rhythm	0.452 (0.261–0.782)	0.005
Out-of-hospital cardiac arrest	3.114 (1.401–6.923)	0.005
CPR to ECMO pump-on time (min)	1.036 (1.023–1.050)	<0.001
CRRT	1.880 (1.073–3.293)	0.027
HH	1.955 (1.048–3.647)	0.035

ECPR, extracorporeal cardiopulmonary resuscitation; CPR, cardiopulmonary resuscitation; ECMO, extracorporeal membrane oxygenator; CRRT, continuous renal replacement therapy; HH, hypoxic hepatitis; * adjusted with gender, malignancy, dyslipidemia, chronic kidney disease, chronic liver disease, and ECMO duration.

## Supplementary Materials

The following are available online at https://www.mdpi.com/2077-0383/9/9/2994/s1, Table S1: Results of the liver tests according to the time of ECPR, Table S2: Incidences of complications associated with ECMO.

## Abbreviations

AST	aspartate aminotransferase;
ALT	alanine aminotransferase;
CPR	cardiopulmonary resuscitation;
CRRT	continuous renal replacement therapy;
ECMO	extracorporeal membrane oxygenation;
ECPR	extracorporeal cardiopulmonary resuscitation;
HH	hypoxic hepatitis;
INR	international normalized ratio.
