# Supplementary material for: The Impact of Hypoxic Hepatitis on Clinical Outcomes after Extracorporeal Cardiopulmonary Resuscitation
Source: J Clin Med. 2020 Sep 16;9(9):2994. doi: 10.3390/jcm9092994 (PMC7565649; doi:10.3390/jcm9092994)
Supplement: Supplementary file 1 [file jcm-09-02994-s001.pdf]

Table S1. Results of the liver tests according to the time of ECPR.

	HH (*n*=90)			
	**Baseline**	**Day 0**	**Day 1**	**Day 2**
AST, U/L	31.0 (20.0–70.0) (*n* = 57)	147.0 (67.0–394.0) (*n* = 89)	322.5 (139.3–757.8) (*n* = 78)	215.5 (91.0–464.0) (*n* = 69)
ALT, U/L	24.0 (16.0–48.0) (*n* = 57)	82.0 (37.0–227.0) (*n* = 89)	116.0 (53.0–282.8) (*n* = 78)	93.5 (44.8–245.8) (*n* = 69)
ALP, U/L	76.5 (58.0–102.8) (*n* = 54)	71.0 (50.8–103.3) (*n* = 81)	54.0 (42.0–77.0) (*n* = 73)	55.0 (42.5–72.5) (*n* = 66)
LDH, IU/L	928.0 (641.5–1499.5) (*n* = 78)	1446.0 (920.0–2612.5) (*n* = 25)	1851.0 (1147.5–3437.5) (*n* = 32)	2187.0 (1549.3–4036.5) (*n* = 12)
INR	1.08 (1.0–1.3) (*n* = 39)	1.9 (1.4–2.9) (*n* = 84)	1.6 (1.3–2.1) (*n* = 75)	1.4 (1.2–1.7) (*n* = 69)
Total bilirubin, mg/dL	0.7 (0.5–1.2) (*n* = 57)	1.0 (0.6–1.6) (*n* = 89)	1.5 (1–2.5) (*n* = 77)	1.7 (1.0–2.8) (*n* = 68)
Albumin, g/dL	3.9 (3.5–4.3) (*n* = 56)	2.7 (2–3.4) (*n* = 86)	2.8 (2.4–3.2) (*n* = 76)	2.9 (2.6–3.1) (*n* = 63)

Numbers are median (interquartile range); AST, aspartate aminotransferase; ALT, alanine aminotransferase; ALP, alkaline phosphatase; LDH, lactate dehydrogenase; INR, international normalized ratio.

Table S2. Incidences of complications associated with ECMO.

	**HH (*n* = 90)**	**Non-HH (*n* = 275)**	***p* Value**
Composite of complications	55	89	<0.001
Limb ischemia	10	18	0.158
Groin hematoma	6	16	0.769
ECMO site bleeding	13	23	0.093
Gastrointestinal bleeding	8	14	0.189
Stroke	6	6	0.038
Sepsis	6	5	0.020
ECMO insertion site infection	3	1	0.019
Rhabdomyolysis	3	6	0.541
In hospital mortality	65	151	0.004
